# Reliable low-temperature die attach process using Ag/Sn/Ag sandwich structure for high-temperature semiconductor devices

**DOI:** 10.1038/s41598-018-37103-7

**Published:** 2019-01-24

**Authors:** Jinseok Choi, Gab Soo Choi, Sung Jin An

**Affiliations:** 1 Department of Advanced Materials Science and Engineering, Kumoh National Institute of technology, 61 Daehak-ro, Gumi-si, Gyeongsangbuk-do 39177 Korea; 2 Fab process engineering department, KEC, 41 Suchul-daero, Gumi-si, Gyeongsangbuk-do 39256 Korea

## Abstract

A low-cost and eco-friendly die attach process for high temperatures should be developed owing to the expansion of the field of high-temperature applications, such as high-power and high-frequency semiconductors. Pb-based and Au-based systems have been used as conventional die attach materials for high-temperature devices. However, these materials exhibit environmental problems and are expensive. Here, we show that the die attach process using the backside metal of the Ag/Sn/Ag sandwich structure is successfully developed for the mass production of Si devices. It has a low-temperature bonding process (235 °C), a high remelting temperature (above 400 °C), and rapid bonding time (20 ms). In addition, it exhibits better properties than Au-12Ge and Pb-10Sn backside metals, which are conventional materials for the high-temperature die attach process. After the die bonding process, various reliability tests of Si devices with the Ag/Sn/Ag backside metal structure were performed.

## Introduction

The demands for high-temperature die attach materials that have been utilized for high-temperature semiconductor devices are increasing because of the expansion of high-power and high-frequency device applications. A die attach process that is part of a device packaging process is extremely important for reliability and environmental stability^1,2^. In particular, die attach materials are an important factor in the die attach process, as they influence the characteristics of the semiconductor device. Au-based (Au-Sn, Au-Si, and Au-Ge) and high Pb-Sn (containing 85–97 wt% Pb) alloys are utilized commercially for high-temperature bonding materials in the semiconductor industry owing to their excellent properties^3–5^. Because Au-based alloys increase the manufacturing cost, they must be replaced by Au-free materials. High Pb-Sn alloys, which are cheap and exhibit good reliability, are excellent materials for high-temperature processes. However, Pb cannot be utilized in electronics because of the restriction of hazardous substances (RoHS) directive.

To replace Pb-based and Au-based materials for the high-temperature die attach process, Bi-based, Zn-based, and Sn–Sb alloys have been studied for high-temperature semiconductor devices^6–12^. Bi-based die attach materials exhibit limited wettability on a Cu lead frame and relatively low thermal and electrical conductivities^6,7^. Zn-based alloys exhibit several drawbacks as high-temperature die attach materials, such as lifespan and poor wetting^4^. Sn–Sb alloys exhibit a stable microstructure and good creep properties^12^. However, they cannot be used for the die attach process, due to the toxicity of Sb. These materials exhibit high melting temperatures and eutectic or near-eutectic compositions, and the die bonding process is conducted at a relatively high temperature (~300 °C). A high bonding temperature may strongly affect the reliability and lifespan of the semiconductor devices because of the increase in the thermal stress between a chip (or a die) and a lead frame caused by the difference in the coefficient of thermal expansion^13^. Transient liquid phase (TLP) bonding and low-temperature sintering are good candidates as a die attach process for high-temperature semiconductor applications; however, the techniques have been studied for interfacial reactions and mechanical properties, and they exhibit long bonding times as long as few minutes^14,15^. Hence, to date, these bonding methods have not been applied for the mass production of semiconductor devices. Therefore, new cheap and eco-friendly high-temperature bonding materials or processes should be developed to increase the fabrication yield and reduce the manufacturing cost of semiconductor devices.

The Sn–Ag binary system is a good candidate for the die attach process and has been used as a conventional low-temperature solder material with a melting temperature lower than 250 °C^16^. Studies on the Sn–Ag binary alloy have primarily focused on the low-melting temperature alloy; few studies on high-temperature die attach materials have been conducted^17^. The backside metallization process is typically used to attach a chip to a lead frame for high-power and high-frequency semiconductor packaging because it exhibits several advantages such as excellent bond-line and good electrical and thermal conduction^18^. Herein, we report a die attach process using the backside metal (BSM) of a Ag/Sn/Ag sandwich structure with a low bonding and high remelting temperature. Our die attach process offers a rapid bonding time (only 20 ms) and is reliable; it is applicable to the mass production of semiconductor packaging.

## Results

**Figure 1:**
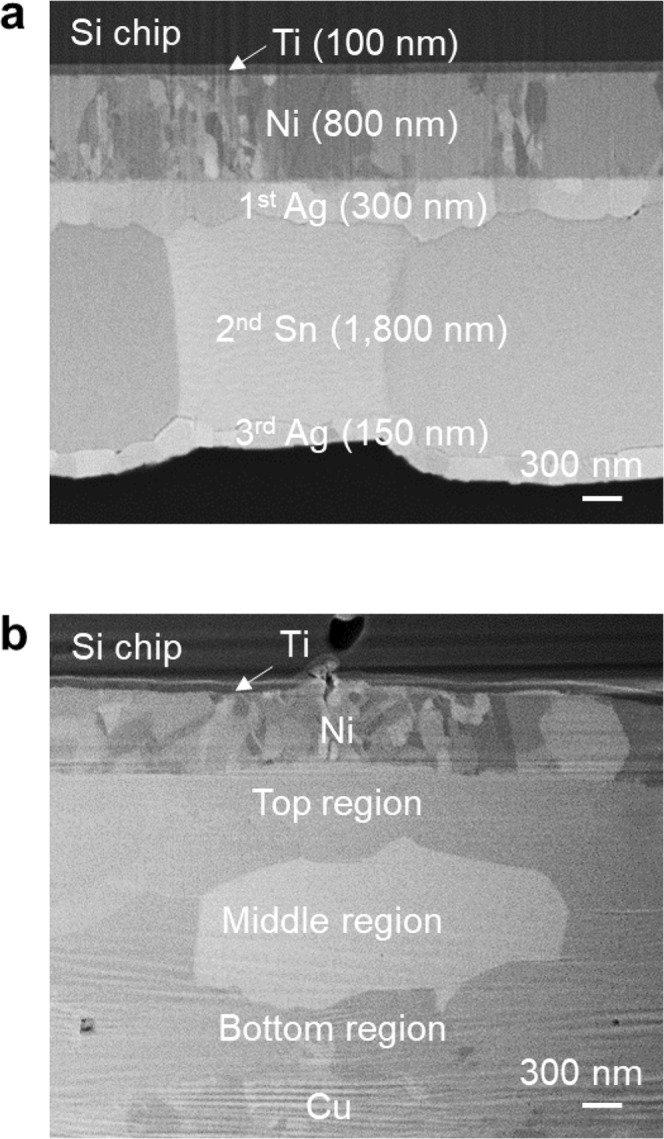
Cross-sectional images of the ASA–BSM layer. Before (**a**) and after (**b**) the die attach process via SEM–FIB. Three regions exist between the Ni layer and the Cu layer after the die attach process.

**Figure 2:**
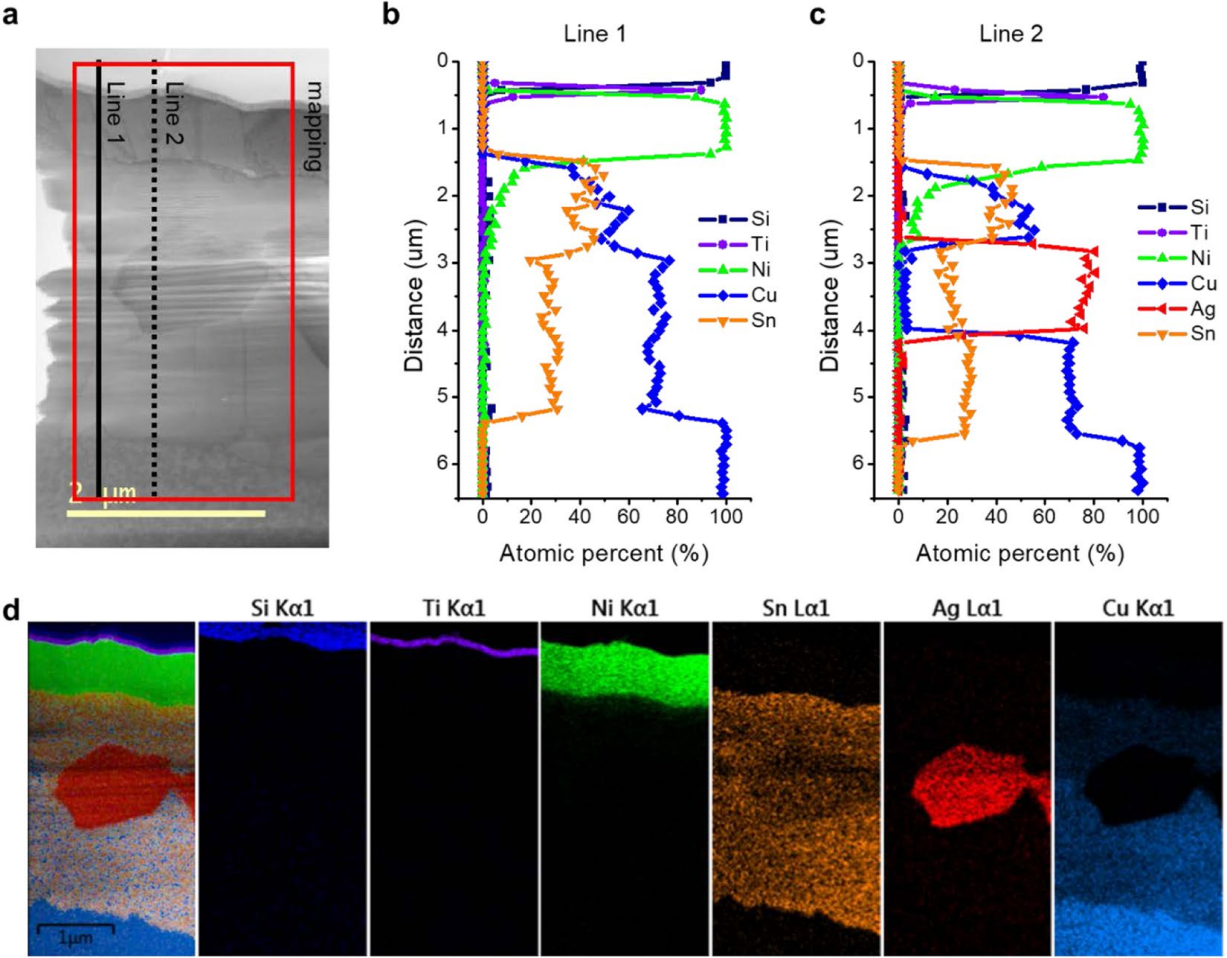
Interface structure between the Si chip and the Cu plated Alloy 42 lead frame. Line (**b**,**c**) and area (**d**) mapping results of the interface between the Si chip and the Cu-plated layer obtained via TEM–EDS.

Figure 1a shows a cross-sectional image of the 1^st^ Ag/2^nd^ Sn/3^rd^ Ag BSM (ASA–BSM) before die attachment. Ti/Ni/1^st^ Ag/2^nd^ Sn/3^rd^ Ag metal layers were formed. The ASA–BSM exhibits a flat surface morphology as shown in Figure S1. After die attachment, three regions exist between the Ni layer and the Cu plating layer, as shown in Fig. 1b (Figure S2 shows the entire bond-line). Figures 2 and S3 show the quantitative elemental analysis of the interface line and area mapping via transmittance electron microscopy–energy-dispersive X-ray spectroscopy (TEM–EDS), respectively. As shown in Figure S3, on average, Cu 50.1 at%, Sn 45.6 at%, and Ni 4.3 at% in the top region; Ag 75.2 at%, Sn 23.3 at%, and Cu 1.5 at% in the middle region; Cu 73.46 at%, Sn 26.54 at%, and 0.9 at% Ag in the bottom region were detected. Interestingly, the high Cu content exceeded the content of Sn and Ag in the top region. In addition, the Ag-rich region (middle region) was formed at the interface. These results will be discussed later. Further structural characterization of the interface was performed via high-resolution TEM. As shown in Figure S3, Cu_6_Sn_5_ in the top region (close to the Si chip), Ag_3_Sn in the middle region, and Cu_3_Sn in the bottom region (close to the lead frame) were confirmed from selected-area electron diffraction (SAED) patterns and EDS results. The ASA–BSM might exhibit a high remelting temperature after the die attachment owing to the interfacial structure composed of Cu_6_Sn_5_ (415 °C), Cu_3_Sn (676 °C), and Ag_3_Sn (480 °C) intermetallic compounds (IMCs)^19,20^.

**Figure 3:**
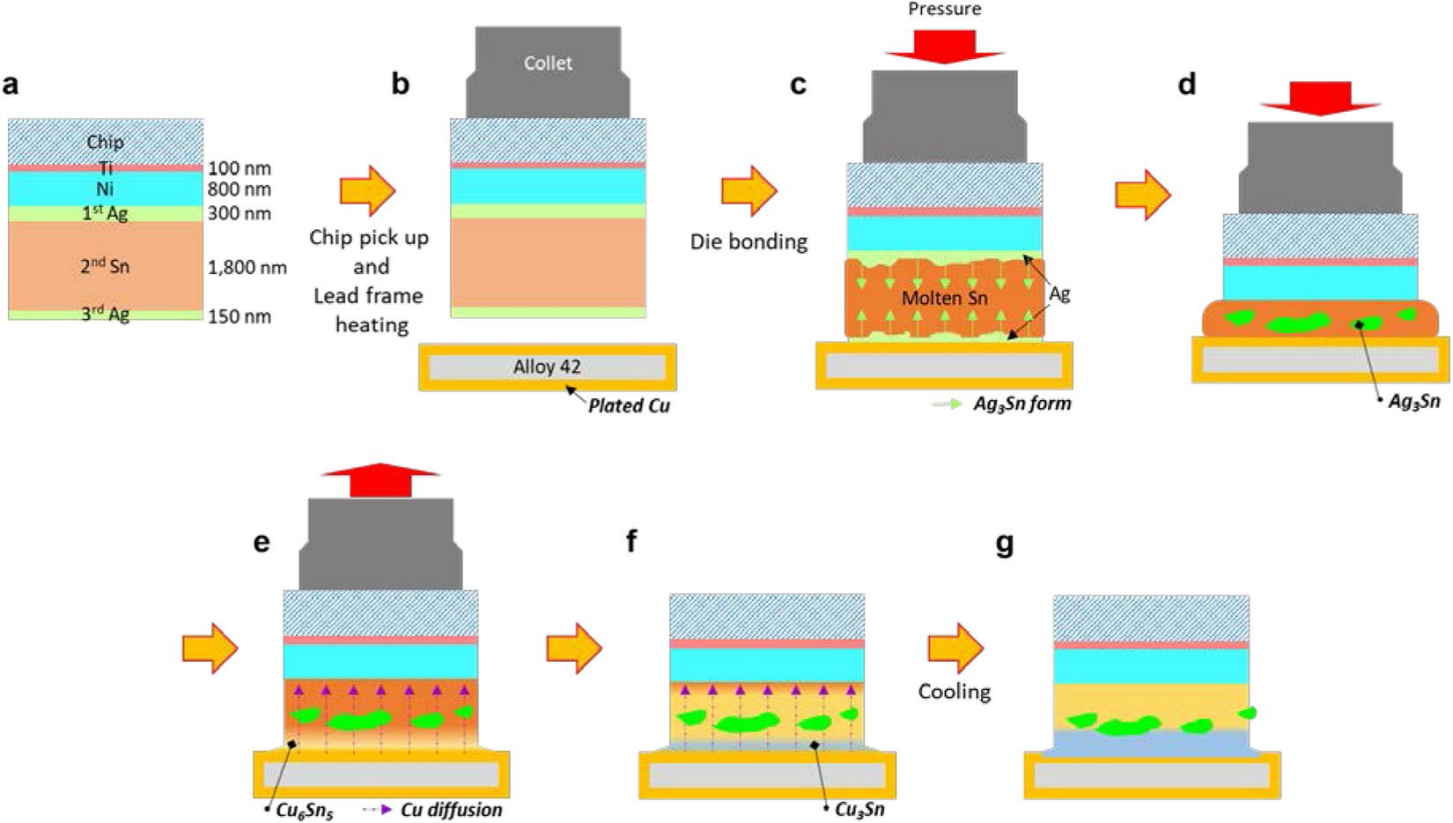
Schematics of the interface formation of the ASA–BSM between the Si chip and the Cu-plated Alloy 42 lead frame. (**a**) BSM layers deposited on the backside of a Si chip. (**b**) Si chip is picked up by a collet. The lead frame was heated to 370 °C. (**c**) Si chip attached to the lead frame. The Sn was melted, and the reaction of the molten Sn and the Ag layers was started. (**d**) Ag_3_Sn IMCs were formed via the continuous reaction of molten Sn and both Ag layers. The Ag_3_Sn IMCs moved to the middle of the molten Sn owing to the pressure during the die attachment. (**e**) After the dissolution of the 3^rd^ Ag layer, the reaction of the Cu and the molten Sn was started, similar to the formation of the Ag_3_Sn. (**f**) Cu_6_Sn_5_ and Cu_3_Sn were grown continuously in the direction of the violet arrows. (**g**) After the die bonding process, the interface was composed of Ag_3_Sn, Cu_6_Sn_5_, and Cu_3_Sn.

We assumed two possible mechanisms for the interface formation during the die attach process. The first is the epitaxial growth of the Cu_3_Sn, Cu_6_Sn_5_, and Ag_3_Sn IMCs, as heat was transferred from the lead frame to the Si chip. However, the BSM consisted of polycrystalline Cu_6_Sn_5_, Cu_3_Sn, and Ag_3_Sn IMCs, separately; additionally, a grain boundary of the IMCs was identified between the Cu_3_Sn and Ag_3_Sn, as shown in Figs 2 and S4. The second is that Ag_3_Sn IMC was formed initially; subsequently, Cu was diffused and Sn–Cu IMCs (Cu_6_Sn_5_ and Cu_3_Sn) were grown. This matches well with our experimental results. Figure 3 shows the schematics of the bonding process using the ASA–BSM structure between the Si chip and the Alloy 42 lead frame. First, the Ti/Ni/Ag/Sn/Ag metal layers were deposited on the backside of the Si chip, as shown in Fig. 3a. At this stage, Ag_3_Sn IMCs could be formed partially after the deposition of the 3^rd^ Ag layer, because Ag–Sn is one of the fast interdiffusion couples with a mean interdiffusion coefficient of 10^−13^ cm^2^/s at room temperature^21,22^. Subsequently, as shown in Fig. 3b, the Si chip is picked up by a collet, which is a part of the die bonder, to attach the chip to the lead frame. The lead frame was preheated to 370 °C before the attachment of the Si chip. Figure 3c shows the Si chip attached to the Alloy 42 lead frame with a bonding force of 0.1 N for 20 ms. The lead frame temperatures were 235–400 °C. When the lead frame was heated to the die bonding temperature, the melting of the Sn layer was started from the Sn–Ag eutectic point (221 °C). At this stage, Ag_3_Sn was formed and grown via the reaction of the 1^st^ and 3^rd^ Ag layers and the molten Sn. The Ag_3_Sn grew via the continuous supply of Ag from the Ag layers through the grown Ag_3_Sn layers (Fig. 3c)^23^. Therefore, the observation of the Ag_3_Sn IMC and the high Ag content in the middle region after the die bonding of the ASA–BSM may have caused the preferential formation of the Ag_3_Sn IMC at the interface of the Ag and the Sn before the reaction of Cu and Sn. Furthermore, the Ag_3_Sn IMCs moved to the middle of the molten Sn owing to the pressure during the die attachment (Fig. 3d). After the dissolution of the 3^rd^ Ag layer, the reaction of the Cu and the molten Sn were started, similarly to the formation of Ag_3_Sn, as shown in Fig. 3e. Cu_6_Sn_5_ IMCs were formed via the reaction of the Cu plating layer and molten Sn. The Cu_6_Sn_5_ and Cu_3_Sn IMCs grew continuously in the direction of the violet arrows while the temperature of the lead frame was decreased to room temperature (Figure 3f,g). Cu_6_Sn_5_ IMC formation is possible within only 5 s at 280 °C at the Sn–Ag/Cu interface^24–26^. Hence, Cu–Sn IMCs could be grown at the interface between the Si chip and the Alloy 42 lead frame. Figure 3g shows the interface structure with three regions consisting of Ag_3_Sn, Cu_6_Sn_5_, and Cu_3_Sn after our die bonding process. This die attach process using the ASA sandwich structure is highly similar to the solid–liquid interdiffusion (SLID) bonding, which is also called transient liquid-phase bonding. SLID bonding has been typically studied for the joining of high-temperature semiconductor devices^27–29^. The benefits of SLID bonding are the low bonding temperature near the melting point of the low-melting point bonding metal and high-temperature stability after the bonding process. In addition, it can form an excellent bond-line and exhibits good gap-filling capability^30–32^. Meanwhile, the most pressing problem in SLID bonding is the long bonding time, which can reach a few minutes for forming a stable bond-line^28^. Hence, to date, SLID bonding has not been applied for the mass production of semiconductor devices. However, this disadvantage was not observed in our experiment, owing to the optimized BSM structure and the die attach process. That is, our die attach method with the ASA sandwich structure involved a low bonding temperature (235 °C), a high remelting temperature, and rapid bonding time (20 ms).

**Figure 4:**
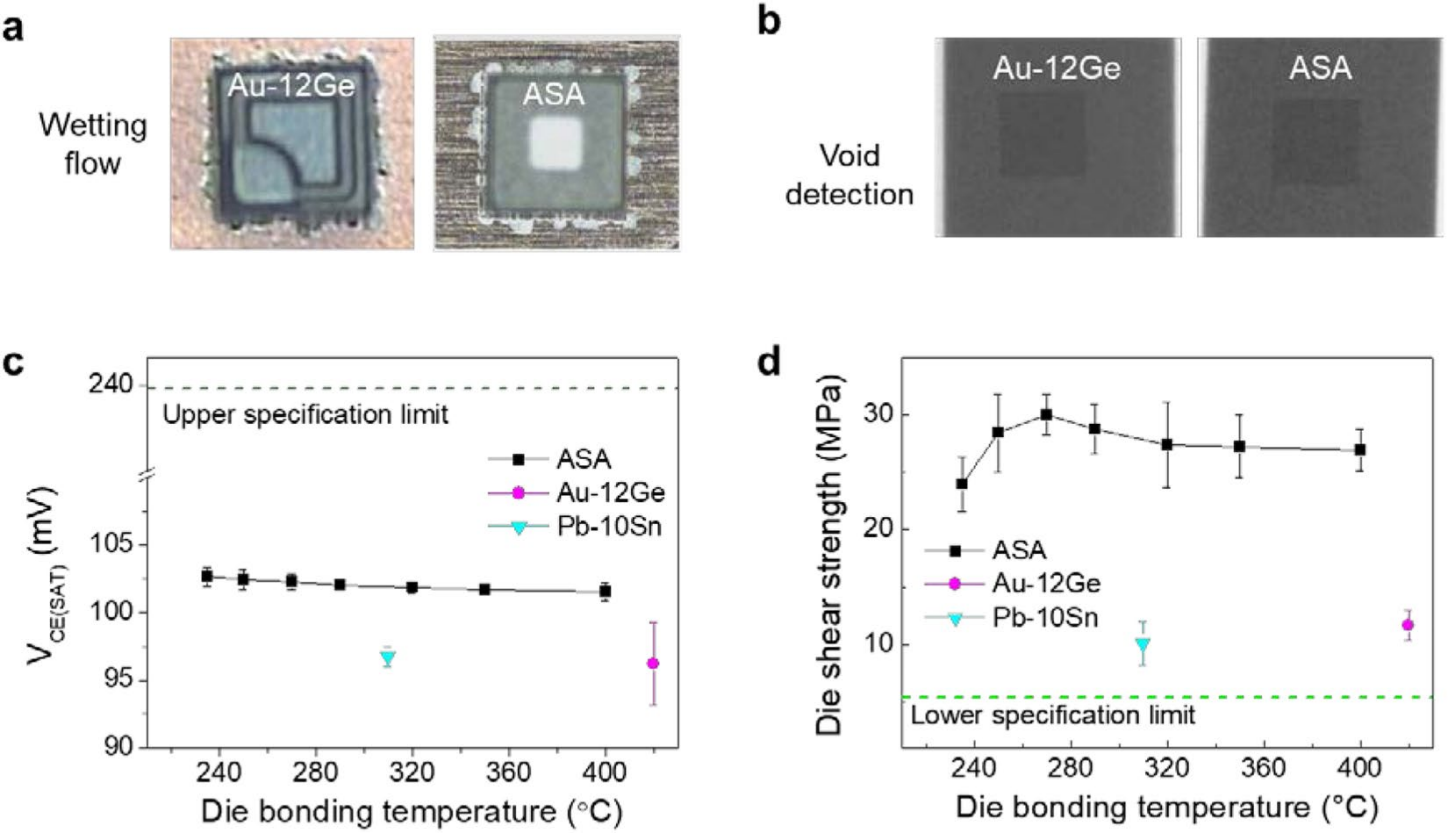
Characterization after the die bonding of the Si chips with the ASA–BSM. (**a**,**b**) Wetting flow images (**a**) and X-ray images (**b**) of Si devices with Au-12Ge and ASA–BSM, obtained after the die bonding. The Au-12Ge BSM is a conventional Pb-free BSM. (**c**) V_CE(sat)_ values of the ASA, Au-12Ge, and Pb-10Sn BSMs depending on the die bonding temperature. The die bonding temperatures of the Au-12Ge and Pb-10Sn BSMs were 400 and 310 °C, respectively. (**d**) Die shear strength of the ASA–BSM depending on the die bonding temperatures.

The various characteristics of the ASA–BSM were evaluated in comparison with Au-12Ge BSM, which is currently used as a conventional BSM, as shown in Fig. 4. Figure 4a,b show the wetting flow and X-ray images of the ASA and Au-12Ge BSMs after the die attach process, respectively. As shown in Fig. 4a, the Si chips with the ASA–BSM exhibited an excellent wetting property. In addition, the X-ray images demonstrate the excellent bonding property; few voids were formed in the ASA–BSM, as shown in Fig. 4b. The voids and wetting property of the ASA–BSM are comparable with those of the Au-12Ge BSM. Figure 4c shows the collector–emitter saturation voltage (V_CE(sat)_) of the ASA and Au-12Ge BSM bonded devices under various conditions. The V_CE(sat)_ values of the ASA–BSM are lower than the upper specification limit, which is for KTC 3875 product (SOT-23 package), and the average V_CE(sat)_ was 102 mV. Figure 4d shows the die shear strength when the die bonding was conducted at different temperatures ranging from 235 to 400 °C with thicknesses of 300 and 150 nm for the 1^st^ and 3^rd^ Ag layers, respectively. The average die shear strength was 26 MPa. When the die attach temperature was below 270 °C, the temperature was lower, and the wetting flow was the worst. As shown in Figure S5, we confirmed the poor wetting flow at the die attach temperature of 235 °C. Meanwhile, when the die attach temperature was 270 °C, the wetting flow was excellent, similar that when the die attach temperature was over 270 °C. When the die attach temperature was over 270 °C, the bulk intermetallic compound (Ag_3_Sn, Cu_6_Sn_5_, and Cu_3_Sn) sizes may affect the die shear strength^33^. At all die attach temperatures, the die shear strengths were larger than the lower specification limit of 5 MPa. In addition, regardless of the die attach temperatures, the die shear strengths of all samples were larger than those of the Au-12Ge BSM with 11 MPa.

We conducted various reliability tests to ensure the stable quality of the die attach process using the ASA–BSM. The lifespan of 44 packages was evaluated via a steady-state operational life (SSOL) test and a high-humidity high-temperature reverse-bias (H3TRB) test. In addition, the thermal stability of 175 packages was evaluated via a solder heating test (SHT), thermal cycle test (TCT), and thermal fatigue test (TFT). After the reliability tests, the performance of all samples was retained, as shown in Supplementary Table 3. The results prove that the ASA–BSM can be applied for semiconductor packaging.

## Conclusion

We demonstrated a low-cost and eco-friendly die attach process using the ASA-BSM structure for fabricating semiconductor devices. Si chips with the ASA–BSM were successfully bonded on an Alloy 42 lead frame with a low bonding temperature (235 °C), high temperature stability, and rapid bonding time (20 ms). In addition, the ASA–BSM exhibited better characteristics than conventional BSMs (Au-12Ge and Pb-10Sn) and passed various reliability tests. The results proved that the ASA–BSM can significantly reduce the manufacturing costs and increase the reliability and yield of device production. Additionally, we began mass producing Si devices with the ASA–BSM without any problems since 2014. Thus far, approximately 1 billion packages such as SOT-23, SOT-23(1), and TO-92 have been produced. More generally, we believe that the ASA–BSM can be readily expanded to fabricate many other semiconductor devices.

## Methods

### Backside metallization

First, the backside native oxides of patterned Si wafers (transistors and diodes) were removed in 1% hydrofluoric acid (HF) solution for 5 s. Next, the wafers were placed in deionized water for 20 min to remove the 1% HF solution. Finally, the wafers were dried at 80 °C for 20 min in a drying oven. After removing the native oxides, Ti (100 nm)/Ni (800 nm)/1^st^ Ag (300 nm)/2^nd^ Sn (1,800 nm)/3^rd^ Ag (150 nm) layers were evaporated on the backside of the Si wafers using a four-pocket e-beam evaporator (CHA Industries, Mark 50) at 2 × 10^−6^ Torr. The deposition rates of the Ti layer and the other layers (Ni, Ag, and Sn) were 0.6 and 0.8 nm/s, respectively. The Si wafer temperatures during the evaporation of Ti, Ni, 1^st^ Ag, 2^nd^ Sn, and 3^rd^ Ag were maintained at 170, 140, 70, 60, and 50 °C, respectively. In the experiment, the Ti layer was an adhesion layer and the Ni layer was a diffusion barrier layer.

### Die attach process

After the deposition of BSM layers on the backside of the Si wafer, wafer dicing was conducted to produce Si chips of size 0.29 mm × 0.29 mm. The Si chips were attached to the Cu-plated Alloy 42 (Fe-42 wt% Ni, Cu thickness is 5 μm) lead frame using a die bonding equipment (TSP, KD-530) for 20 ms, which is the conventional die bonding time for Si device fabrication. The die bonding force was 0.1 N. The lead frame temperatures were varied to determine the lowest die bonding temperature and evaluate the mechanical and electrical properties in the temperature range of 235 to 400 °C.

### Characteristics

After the BSM deposition, the surface morphology and cross section of the BSM stack on Si were investigated via scanning electron microscopy (SEM, FEI, Nova 600 NanoLab). Moreover, a focused ion beam (FIB) was used to investigate the cross section of the BSM layers. The change in the cross-section of the BSM layer after the die bonding process was investigated via SEM–FIB (JSM-6500F, JEOL). In addition, field-emission transmission electron microscopy (JEM-ARM200F, JEOL) was used to obtain high-resolution images and selected-area electron diffraction patterns. X-ray inspection was conducted using X-TV 160 (M-Tek) with an X-ray source of 100 KV and 50 μA, and an output power of 20 W. Die shear-strength tests were conducted using a chip of size 0.29 mm × 0.29 mm, and approximately 200 die assemblies were tested. The electrical characteristics were evaluated for 270 pieces using a discrete device test system (TESEC, 971TT). The V_CE(sat)_ values were measured under the condition of I_C_ = 100 mA and I_B_ = 10 mA. The test condition was for the KTC 3875 product (SOT-23 package), which is the epitaxial planar NPN transistor. Lifetime tests (SSOL and H3TRB) and environmental tests (SHT, TCT, and TFT) were conducted for ensuring the reliability using the SOT-23 package, which is a typical package for transistors and diodes. The SOT-23 package fabricated after the Si chip was attached to the Cu-plated Alloy 42 lead frame at temperatures of 270, 300, 320, 360, and 400 °C, respectively. The SSOL test was conducted at 25 °C. The H3TRB test was conducted at 80 °C and 85% humidity. The SHT was conducted at 265 °C for 10 s. For the SSOL test, H3TRB test, and SHT, 44 pieces were used. The TCT was conducted with 200 cycles ranging from −55 to 150 °C for 15 min. The TFT was conducted in the temperature range of 25 to 150 °C with an on-time of 10 s and an off-time of 50 s. For the TCT and TFT, 175 pieces were used.

## Supplementary information

Supplementary information
