# Supplementary material for: Reliable low-temperature die attach process using Ag/Sn/Ag sandwich structure for high-temperature semiconductor devices
Source: Sci Rep. 2019 Jan 24;9:555. doi: 10.1038/s41598-018-37103-7 (PMC6345981; doi:10.1038/s41598-018-37103-7)
Supplement: Supplementary file 1 — Supplementary information [file 41598_2018_37103_MOESM1_ESM.docx]

Supplementary information

**Reliable low-temperature die attach process using Ag/Sn/Ag sandwich structure for high-temperature semiconductor devices**

Jinseok Choi^1^, Gab Soo Choi^2^, and Sung Jin An^1, *^

^1^Department of Advanced Materials Science and Engineering, Kumoh National Institute of technology, 61 Daehak-ro, Gumi-si, Gyeongsangbuk-do 39177, Korea

^2^Fab process engineering department, KEC, 41 Suchul-daero, Gumi-si, Gyeongsangbuk-do 39256, Korea


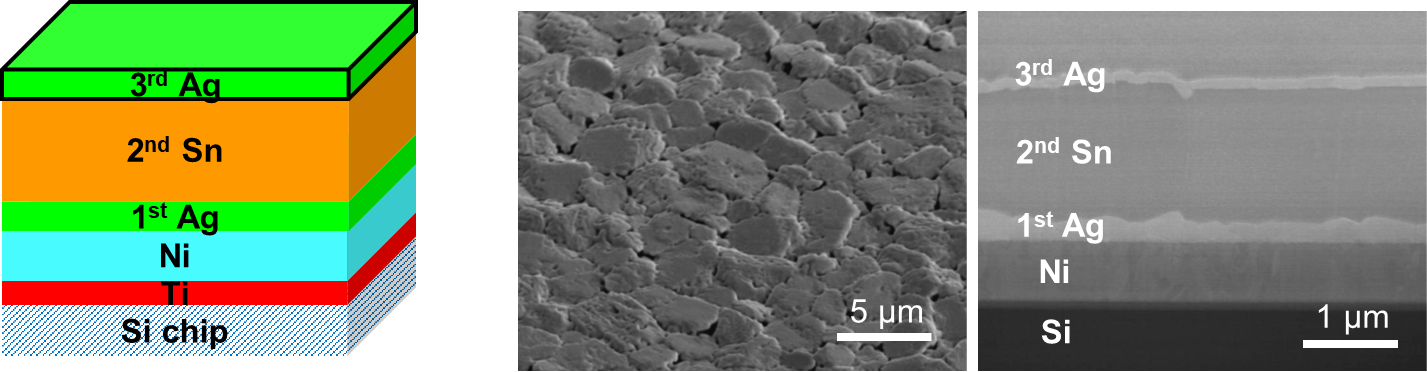


**Figure S1.** Schematics of the BSM structure and SEM images of the surface morphology (bird’s eye view) and cross-section of 1^st^ Ag/2^nd^ Sn/3^rd^ Ag BSM (ASA–BSM) structure. The ASA–BSM structure exhibited a flat surface morphology.


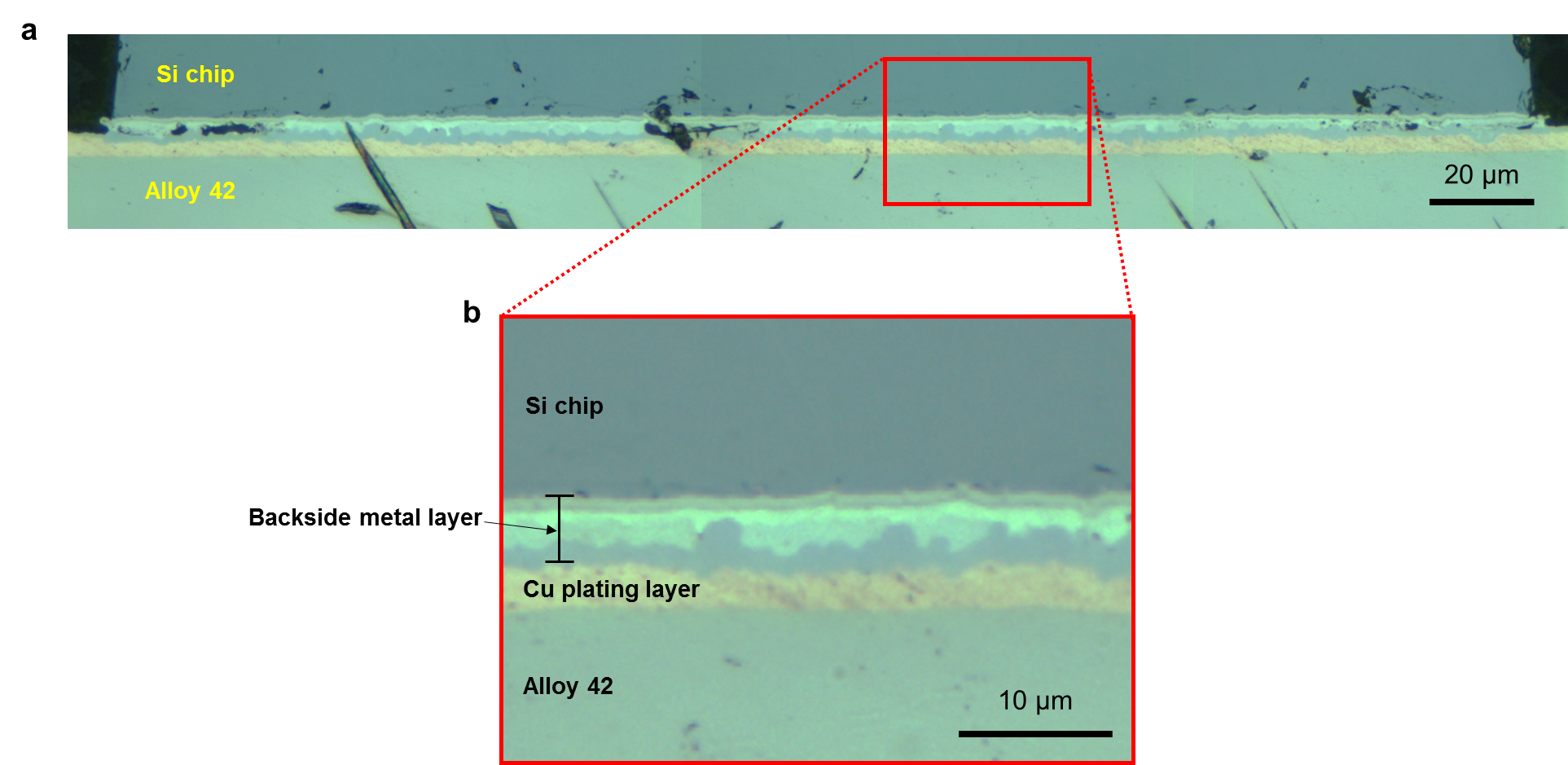


**Figure S2.** Cross-sectional images of the ASA–-BSM after die attachment on the Alloy 42 lead frame. (a) Interface between the Si chip and the Alloy 42 lead frame. (b) Enlarged image of the red-rectangle region in Supplementary Fig. 5a. Voids were not observed between the Si chip and the Alloy 42 lead frame. The Cu plating layer thickness decreased from 5 μm to approximately 3 μm.


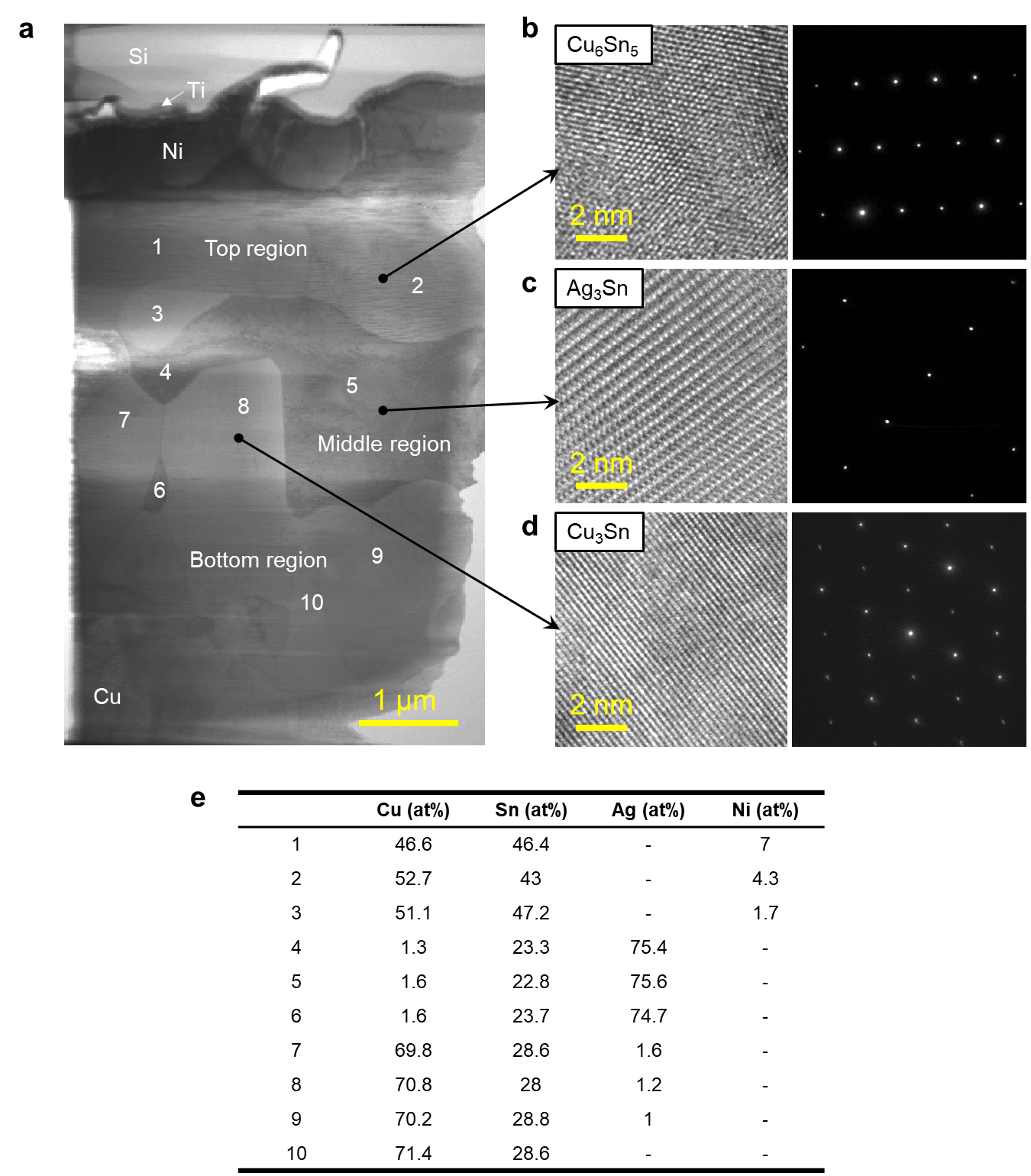


**Figure S3.** (a) TEM image of the ASA–BSM layer after the die attach process. (b–d) HR-TEM images (left) and SAED patterns (right) of the Cu_6_Sn_5_ (b), Ag_3_Sn (c), and Cu_3_Sn (d) IMCs, respectively. (e) Quantitative analysis results for the interface between the Si chip and the Cu plating layer, obtained via TEM–EDS.


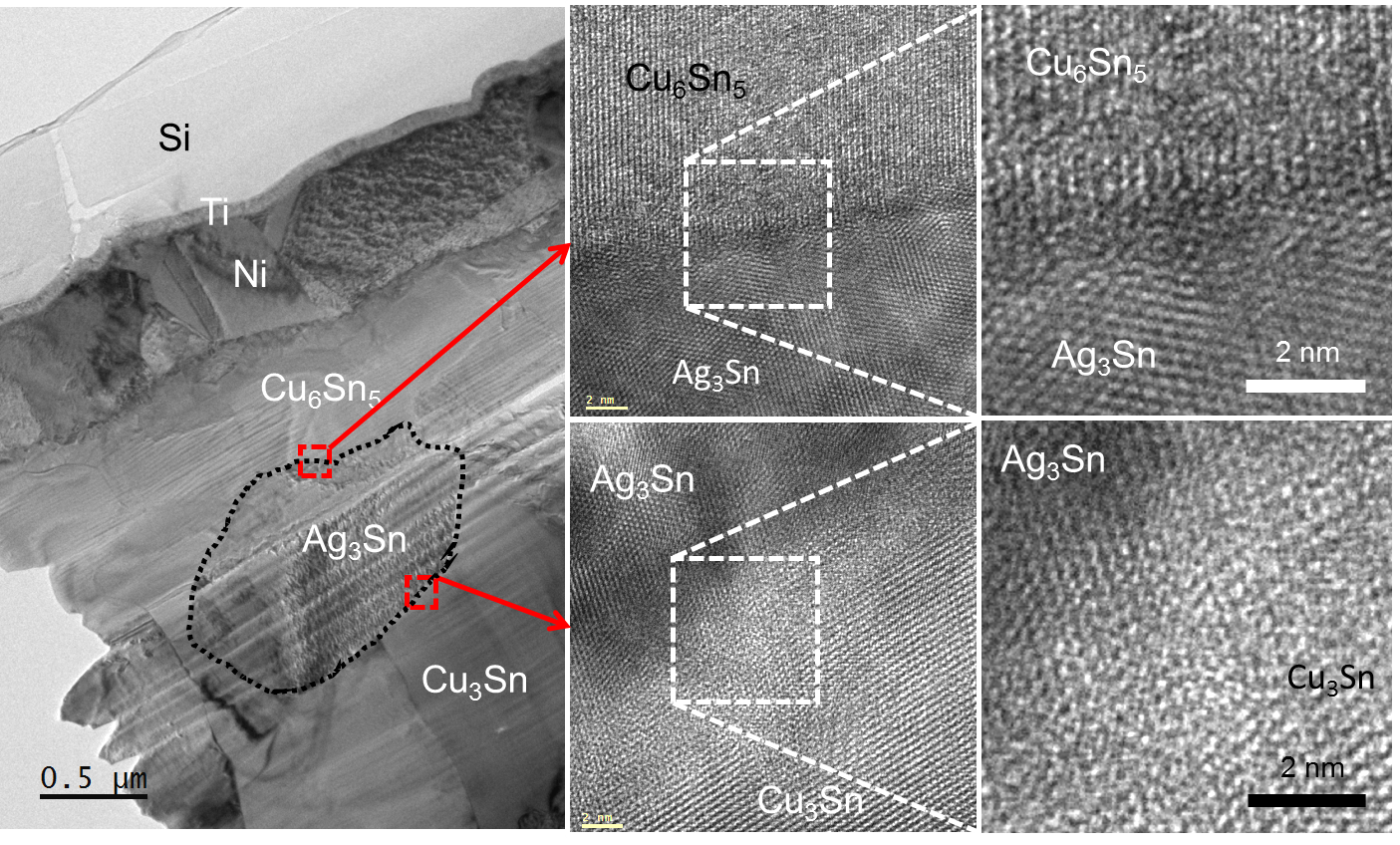


**Figure S4.** HR–TEM images of the Cu_6_Sn_5_/Ag_3_Sn and Ag_3_Sn/Cu_3_Sn interfaces.


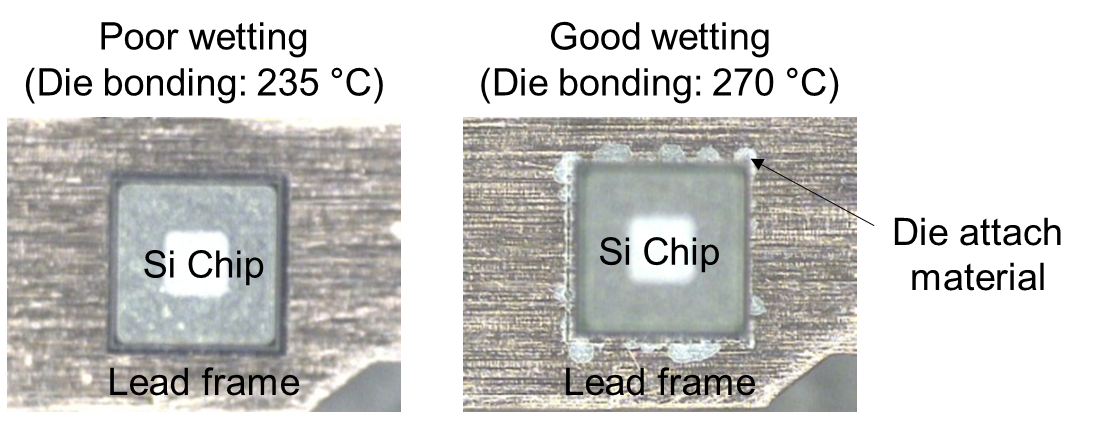


**Figure S5.** Poor (left image) and good (right image) wetting flow images after die attach process with ASA–BSM.

| Reliability test | Au-12Ge | Ag/Sn/Ag | | | | | Results |
| --- | --- | --- | --- | --- | --- | --- | --- |
|  |  | D/B 270 °C | D/B 300 °C | D/B 320 °C | D/B 360 °C | D/B 400 °C |  |
| Steady state operational life (SSOL) | 0/44 | 0/44 | 0/44 | 0/44 | 0/44 | 0/44 | OK |
| High humidity temperature reverse bias (H3TRB) | 0/44 | 0/44 | 0/44 | 0/44 | 0/44 | 0/44 | OK |
| Solder heating test (SHT) | 0/175 | 0/175 | 0/175 | 0/175 | 0/175 | 0/175 | OK |
| Thermal cycle test (TCT) | 0/175 | 0/175 | 0/175 | 0/175 | 0/175 | 0/175 | OK |
| Thermal fatigue test (TFT) | 0/175 | 0/175 | 0/175 | 0/175 | 0/175 | 0/175 | OK |

**Table S1.** Results of several reliability tests of the package using the ASA–BSM.
